# A new concept of drought feeling against the meteorological drought

**DOI:** 10.1038/s41598-022-21181-9

**Published:** 2022-10-06

**Authors:** Farhad Hooshyaripor, Jamshid Sardari, Majid Dehghani, Roohollah Noori

**Affiliations:** 1 Department of Civil Engineering, Science and Research Branch, Islamic Azad University, Tehran, Iran; 2 Technical and Engineering Department, Faculty of Civil Engineering, Vali-e-Asr University of Rafsanjan, Rafsanjan, Iran; 3 Department of Environmental Engineering, Graduate Faculty of Environment, University of Tehran, Tehran, Iran; 4 Faculty of Governance, University of Tehran, Tehran, Iran

**Keywords:** Climate sciences, Hydrology, Natural hazards

## Abstract

Drought is a natural disaster that causes much damage to the communities. Recently, water demand has been increasing sharply due to the population growth and the development process. By approaching the amount of water demand to the natural supplies, any decrease in the water supply may lead to a considerable negative socio-economic consequence. In this condition, the sense of drought prevails over the physical drought. Therefore, usual drought indices can not be used for characterizing and monitoring the drought in a basin. In this paper, multivariate standardized drought feeling index (MSDFI) is introduced which represents two dimensions of water management: (1) water supply in terms of precipitation and (2) water demand in terms of population. The MSDFI is calculated and its variation over time is compared to the standardized precipitation index (SPI). According to the results, MSDFI values in the early years were usually higher than SPI values and vice versa in the last years. This situation is highly correlated with the population trend in the basin. Thereafter, intensity of drought index (IDI) was defined as the difference between MSDFI and SPI to show the role of water demand in the drought feeling. Results show that IDI has an increasing trend in the populated areas, generally downstream of the basin, where population growth is high. In contrast, in the sparsely populated areas generally upstream of the basin where population growth is low and even negative due to migration, the IDI does not show any significant sense of drought.

## Introduction

Drought can be considered as a temporary dry period that lasts long enough to cause imbalances in different hydrological conditions^1^. Drought ranks first among natural disasters in terms of the number of people affected^2^. Also, it is the most damaging natural disaster with vast direct and indirect socio-economic impacts^3^. It can also influence the water resources and ecological safety, such as migration and transformation of the terrestrial ecosystem^4^. The drought phenomenon occurs repeatedly in all types of climates, but its characteristics vary from region to region^5^. Based on the various hydrological cycle deficits, Wilhite and Glantz^6^ classified the droughts into four types (1) meteorological, (2) hydrological, (3) agricultural and (4) socio-economic. A sustained period of meteorological drought may lead to hydrological drought. To provide early warnings of hydrological drought, the propagation threshold from meteorological to hydrological drought is important^7,8^. Moreover, prolonged hydrological drought leads to agricultural and finally to socio-economic drought. Therefore, drought cannot be considered only a natural hydrological phenomenon, but a phenomenon that affects by enhanced water demand. It has been shown that anthropogenic warming and increasing water demands will exacerbate the effects of drought^9^. Smirnov et al.^10^ stated that in the next few decades, water scarcity that happens due to high water use will be a consequence of population growth and climate change. They showed that 61 countries will experience an increase in drought exposure mainly due to population growth alone or interaction between population growth and climate change. By increasing water demand, welfare level, and industrial and agricultural development socio-economic drought becomes a major concern^11,12^.

It is evident that as the water demand increases, the socio-economic drought happens much more rapidly and the time interval between meteorological and socio-economic droughts becomes shorter. Therefore, in the future, we expect to hear more about socio-economic drought and it will be the reason for many social crises. AghaKouchak et al.^13^ claimed that overuse and obsolete management of scarce water resources is exacerbating the impacts of drought wherever population and industries are growing. The population is explicitly incorporated in most of the factors that they considered for drought vulnerability analysis (20 factors out of 26 chosen factors). The drying up of wetlands and lakes, for instance, Urmia Lake^14^, is an indicator that emphasizes the expansion of socio-economic drought in time and space. Up to 2014, about 88% of the Urmia Lake area had been shrunk^14,15^ not only due to the prolonged drought but also to aggressive regional hydro-economic development plans in the Urmia Lake Basin (ULB)^14^. The increasing debates on the drought in communities confirm the hypothesis that the shortening time interval between meteorological and socio-economic droughts depends on the enhanced water demands. Therefore, it is required more comprehensive indices compound not only supply but demand variables to show the reality of drought components in societies. For example, Mehran et al.^16^ developed a supply and demand index, multivariate standardized reliability and resilience index (MSRRI), based on the input–output of reservoirs. Xing et al.^17^ proposed a drought index using the irrigation water supply and demand. Liu et al.^18^ proposed water resources system resilience index (WRSRI) for socio-economic drought analysis. But, a key component ignored in most of these researches is neglecting the role of the population in the exceeding water demand. The main objective of this paper is to introduce a new index for drought monitoring, multivariate standardized drought feeling index (MSDFI), to demonstrate the difference between what happens hydrologically and what people feel in society. Indeed, MSDFI is not a socio-economic drought index. It can be used associated with other drought indices such as standardized precipitation index (SPI) to determine that the socio-economic drought is the result of an imbalance between water supply and demand due to an increase in population or temporary change in the climatic variables such as precipitation.

### Case study area

**Figure 1:**
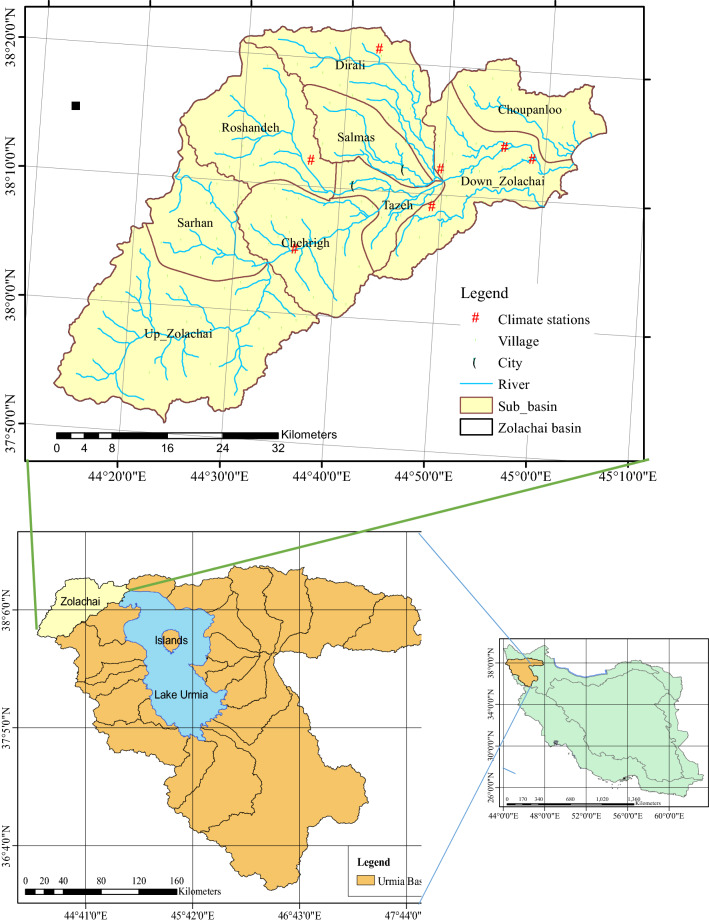
Location of Zolachai basin in ULB and Iran.

**Figure 2:**
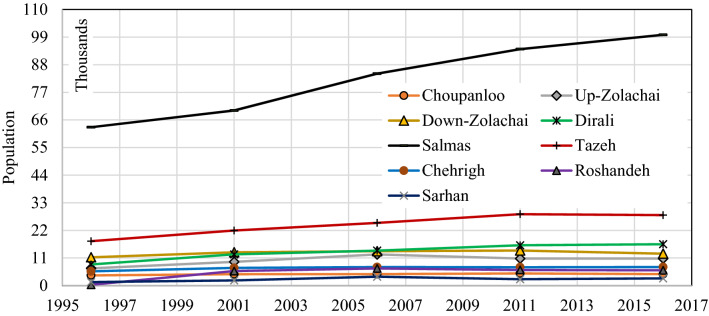
Population trends in different sub-basins.

With a surface area of approximately 5200 km^2^, Urmia Lake is the largest inland lake in Iran and the second largest saline lake in the world^19^. Urmia Lake is a natural water body northwest of Iran (Fig. 1) where rapid unsustainable hydro-economic development has brought several problems to the lake and surrounding basin area. As the natural water supply in the basin has not been sufficient to meet the dramatically increased water demand, the lake has shrunk gradually^14^. In this paper part of ULB, the so-called Zolachai Basin was considered for evaluating the drought feeling using MSDFI. Zolachai Basin lies between longitude 44° 14ʹ and 45° 6ʹ and latitude 37° 53ʹ and 38° 23ʹ in the northwest of the ULB (Fig. 1). This basin has an area of 2259 km^2^ with the highest and lowest points of 2890 and 1260 masl. There are seven meteorological stations (Fig. 1) in the basin: the most important are Karim-Abad, Tapic, Salmas, Abajaloo-Sofla, and Kalhor. Zolachai River, a seasonal river that flows from the Sari Dash mountains around the Kohneh-Shahr city, irrigates the Salmas Plain at the lowest levels of Zolachai Basin and then discharges into the Urmia Lake. Tazeh-Shahr and Salmas cities as well as 139 small cities and villages are located in the basin (Fig. 1). The total population of the basin in 2016 was 188,923 of which 88,000 and 8900 people resided respectively in Salmas and Tazeh-Shahr. Due to the extensive fertile lands in the basin, most people’s occupation is agriculture and the dominant cultivation pattern is the apple orchard. In this research to evaluate and compare the drought in the upstream and downstream points, the Zolachai Basin is divided into 9 sub-basins (Fig. 1). The characteristics of these sub-basins are described in Table 1. Because of the importance of population in this study, the trend of the population in different sub-basins is presented in Fig. 2.

**Table 1:** Characteristics of the sub-basins of Zolachai basin.

Row	Sub-basin	Main river	Area (km^2^)	Annual precipitation (mm)	Important city	Number of villages	Population in 2016	2016 growth rate (%)
1	Choupanloo	Zolachai	166	276	–	6	4576	− 1.12
2	Down-Zolachai	Zolachai	283	270	–	17	12,714	− 1.81
3	Dirali	Diralisoo	153	338.6	–	15	16,490	0.53
4	Salmas	Diealisoo	132	285.6	Salmas	8	99,921	1.21
5	Tazeh	Zolachai	101	259.2	Tazeh-shahr	13	28,078	− 0.27
6	Chehrigh	Zolachai	263	295.2	–	21	7403	0.24
7	Roshandeh	Roshandeh	287	309.6	–	16	6092	− 0.36
8	Sarhan	Sarhan	204	297.6	–	11	2866	2.54
9	UP-Zolachai	Zolachai	581	296.4	–	32	10,783	0.00

**Figure 3:**
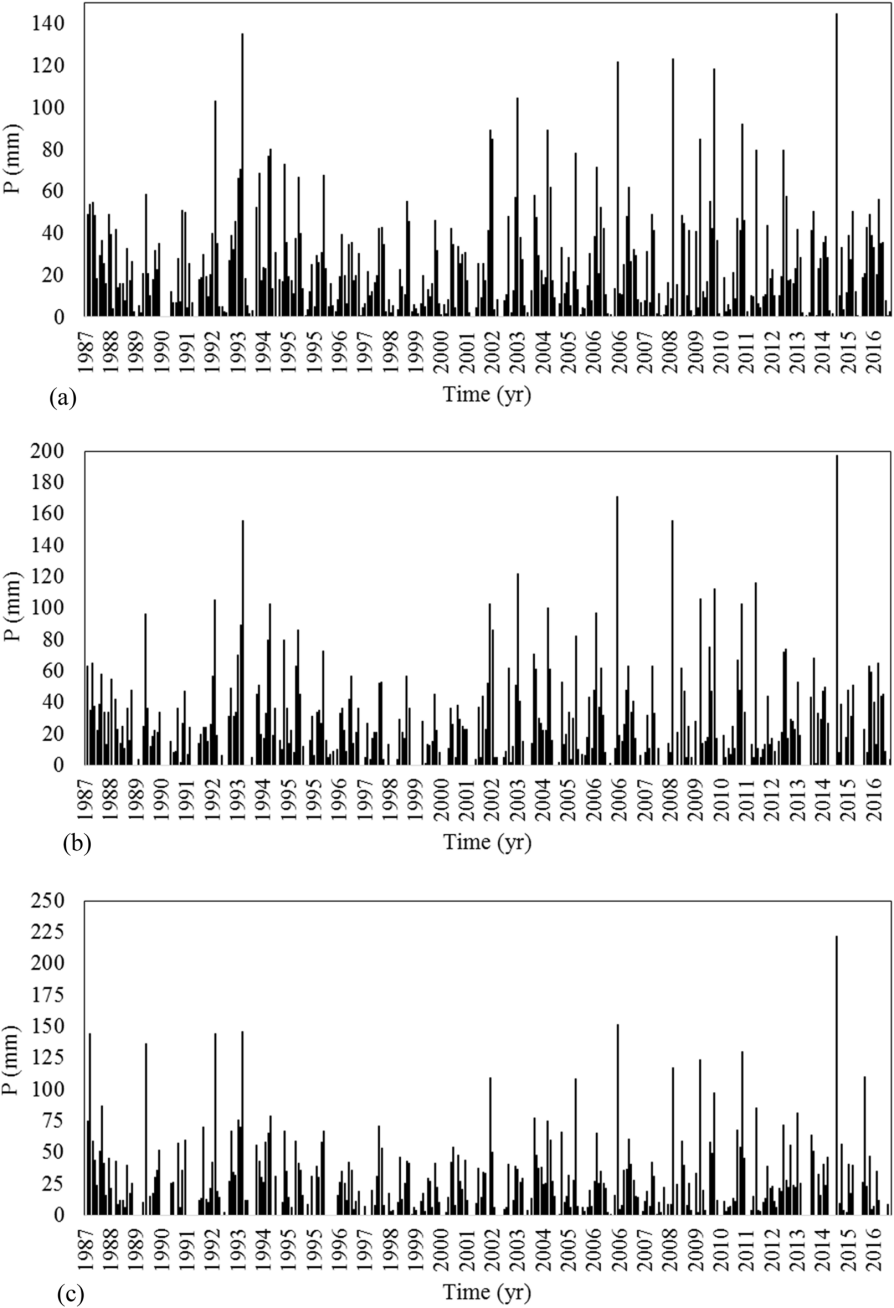
Hyetograph of precipitation in (**a**) Salmas, (**b**) Roshandeh, and (**c**) Up-Zolachai sub-basins.

Among all sub-basins, three of them, including Up-Zolachai (UZSB), Salmas (SSB), and Roshandeh (RSB) were selected for further analysis. These three sub-basins are selected to able us to evaluate the role of population trend upstream where people who live in the low-populated villages usually leave their inhabitants to find better conditions in the populated cities at downstream. Observations in the basin show that there is an increasing trend of immigration from villages to cities besides a decreasing trend in the precipitation. Both phenomena have elaborated the attention to drought and the importance of water management in the Zolachai basin. Salmas with 132 km^2^ area covers about 5.8% of the area of Zolachai basin is located in the central part of the basin and it contains 6 villages out of 139 ones in the basin. Salmas city is also located in this sub-basin. The mean monthly precipitation in the Salmas sub-basin was calculated using the Thiessen method based on three stations around this sub-basin. The hyetograph of monthly precipitation from 1987 to 2016 in SSB was plotted in Fig. 3a. Based upon the precipitation records from 1987 to 2016, the mean annual precipitation in SSB is 285.6 mm. The precipitation follows a seasonal pattern receiving the most precipitation from November to May while there is little precipitation in the rest of the year. The population of SSB was 63,105 people in 1986 while it reached 99,921 people in 2016 with a growth rate of 3.58%.

Roshandeh with a 287 km^2^ area covers 12.7% of the Zolachai basin. It is located in the northwest of Zolachai basin and 16 villages are located in this sub-basin. The monthly precipitation of RSB from 1987 to 2016 is plotted in Fig. 3b. Roshandeh River is the main river in this sub-basin. The mean annual precipitation in RSB is 309.6 mm with the seasonal pattern as described for SSB. The population of this catchment in 1986 was 326 people which increased rapidly to 6814 in 2006 and then decreased to 6092 people in 2016. On the other hand, Up-Zolachai at the highest point of Zolachai basin has a 581 km^2^ area. The average annual precipitation of UZSB from 1987 to 2016 is calculated as 296.4 mm and the sub-basin includes 32 villages.

## Methodology

The SPI is one of the most commonly used indices for drought assessment. SPI was firstly presented by Mckee et al.^20^ for drought monitoring in the state of Colorado, USA. SPI is a multipurpose index and can be used at different timescales (1 to 24 months or more). SPI is based on the calculation of rainfall probabilities in the given timescale. Fitting an appropriate probability density function (PDF) to the precipitation data is the first step in calculating the index. According to Edwards and Mckee^21^, the gamma distribution fits the monthly rainfall data well, so the SPI theory was initially based on this distribution. The gamma PDF is defined as follows:

1 $$ f(x) = \frac{{(x/\beta )^{\alpha - 1} \exp ( - x/\beta )}}{\beta \Gamma (\alpha )},\,\,x,\alpha ,\beta > 0, $$

where *α* and *β* are shape and scale parameters, respectively, *x* is precipitation amount, and Γ(*α*) is the gamma function. Then regarding the standard normal distribution, the standardized precipitation value corresponding to the cumulative probability of gamma distribution function is determined, which is the SPI value.

To calculate MSDFI, at first, a new indicator of per capita precipitation (PCP), is defined as monthly precipitation dividing by the population of the basin. This conversion is also used by Tsakiris et al.^22^ who proposed the ratio of precipitation to potential evapotranspiration as a new parameter for drought analysis that accounts for global warming processes. Then, similarly the approach for calculating the SPI was employed to calculate the MSDFI. In this regard, time series of PCP in different sub-basins were calculated by dividing the precipitation data by the corresponding population data. Then the Kolmogorov–Smirnov (KS) test was used to identify the best PDF of the PCP data (here Gen-Logistic). The KS statistic (D_max_) is the largest vertical difference between the theoretical and the empirical cumulative distribution function:

2 $$D=\underset{1\le i\le n}{\mathrm{max}}\left\{F\left({x}_{i}\right)-\frac{i-1}{n},\frac{i}{n}-F\left({x}_{i}\right)\right\},$$

where $F\left({x}_{i}\right)$ is the probability of ${x}_{i}$ on the cumulative distribution function (CDF), *i* is the rank of ${x}_{i}$, and *n* is the number of data. The null and the alternative hypotheses in the KS test are:

- *H*_0_: the data follow the specified distribution;
- *H*_*A*_: the data do not follow the specified distribution.

If the test statistic, *D*_*max*_, is greater than the critical value (*D*_*c*_), the hypothesis regarding the distributional form is rejected at the chosen significance level (here *α* = 0.05). Finally, Z value corresponding to the cumulative probability of the well-fitted distribution in the standard normal distribution was derived which is the MSDFI value. Based on the MSDFI values and regarding Mckee et al.^20^, drought feeling severity can be classified into five classes as presented in Table 2.

**Table 2:** SPI^20^ and MSDFI drought classification.

No.	Drought class	Index (SPI/MSDFI) range
1	Non-drought	Index ≥ 0
2	Near normal	− 1 < Index < 0
3	Moderate	− 1.5 < Index ≤ − 1
4	Severe	− 2 < Index ≤ − 1.5
5	Extreme	Index ≤ − 2

The difference between the SPI and MSDFI shows the Intensity of Drought Feeling (IDF) and can be used to interpret the sense of drought at any time.

3 $$ \,IDF_{t} = SPI_{t} - MSDFI_{t} \,\,\,\forall \,\,\,t \in T. $$

IDF shows the difference between the meteorological drought and the drought that the community feels. Negative values of IDF mean that the natural drought is dominant and drought feeling is not significant. Therefore, the inhabitants have no considerable drought feeling. Although they are in a drought condition, there is still enough water for consumption and enough water to satisfy the demands with high reliability. In such a condition, meteorological drought is not of so much concern, thus a set of low-level strategies may be needed to pass the drought period. On contrary, a positive values of IDF depict that the sense of drought is much more than the meteorological drought. It means that in such conditions, drought threats the community’s sustainability even though the physical drought severity is not high. As much as the IDF is more positive, the shorter time interval between meteorological drought and socio-economic drought is expected. Hence, to manage the drought more effectively, harder adaptation strategies are needed.

## Results

**Figure 4:**
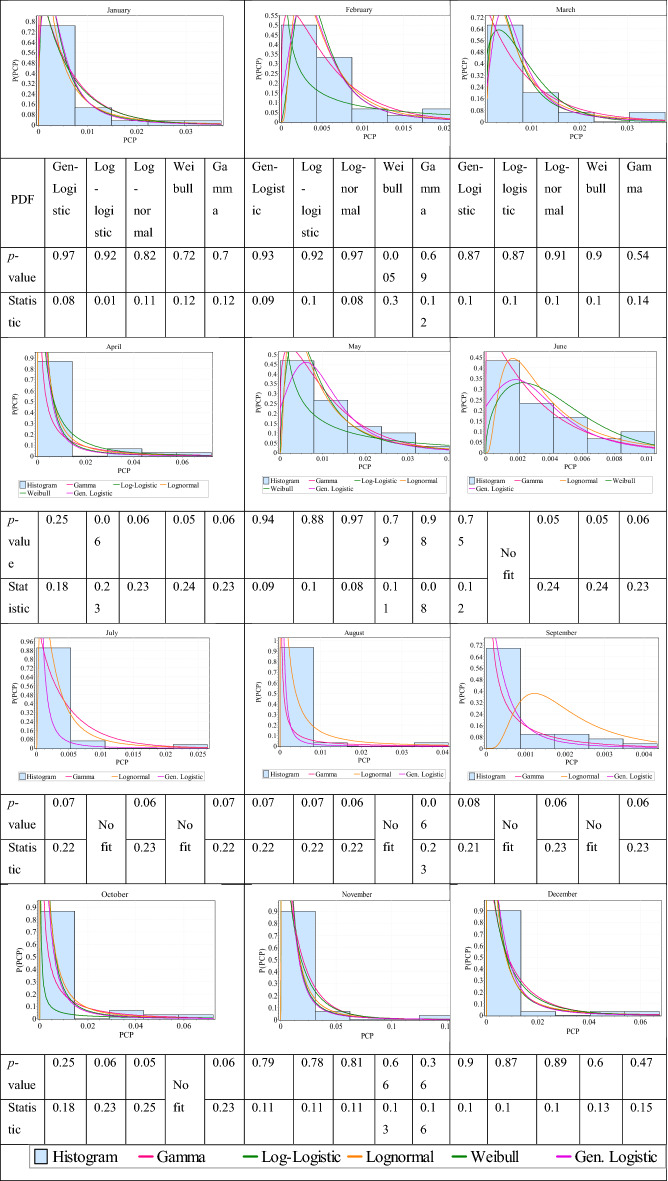
Different PDFs on the PCP values of various months for SSB.

As mentioned previously, the main purpose of this study is to develop a new drought index for drought feeling which considers the water demand in addition to the precipitation. Therefore, in this section, the drought condition will be evaluated by the best analytical probability distribution. In Fig. 4, the fit of five PDFs, including Generalized-Logistic (Gen-Logistic), Log-Logistic, Log-Normal, Weibull, and Gamma associated with the *p*-value and critical statistics for each PDF were illustrated for SSB. As the length of records is 30 years, *D*_*c*_ = 0.24 based on the KS test. Therefore, every PDF with statistics less than *D*_*c*_ will be more suitable for drought analysis and the PDF with the least statistic value is the best. According to Fig. 4, Weibull probability distribution has not been suitable in most of the cases at the 0.05 significance level. By evaluating the fits in each month, it can be concluded that Gen-Logistic is the best fit in cases of statistics and *p*-values. Therefore, Gen-Logistic is considered as the best fit for PCP, then the MSDFI was calculated based on this PDF. Also, for other sub-basins the best PDF was Gen-Logistic.

Then, as described in the methodology, MSDFI is calculated as the normal value corresponding to the cumulative probability in the Gen-Logistic distribution. For this purpose, a code was developed in MATLAB. Moreover, SPI was calculated through another available code^23^ to have a comparison between two indices and the calculation of IDF. The SPI and MSDFI calculated in 3, 6, and 12 aggregated months are called hereafter SPI3, SPI6, SPI12, MSDFI3, MSDFI6, and MSDFI12.

### Drought analysis in Salmas sub-basin (SSB)

**Figure 5:**
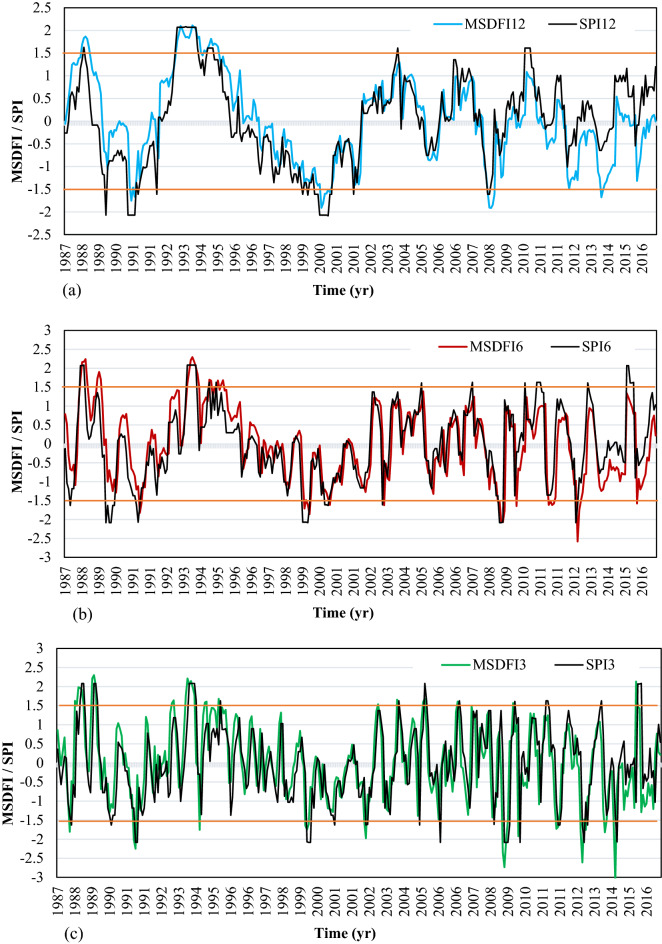
SPI and MSDFI time series in (**a**) 12, (**b**) 6, and (**c**) 3 aggregated months for SSB.

The time series of SPI and MSDFI in different time scales were calculated for SSB and plotted in Fig. 5a–c. Also, the lines corresponding to the $SPI = \pm 1.5$ and $MSDFI = \pm 1.5$(severe drought and wet condition) were plotted.

**Figure 6:**
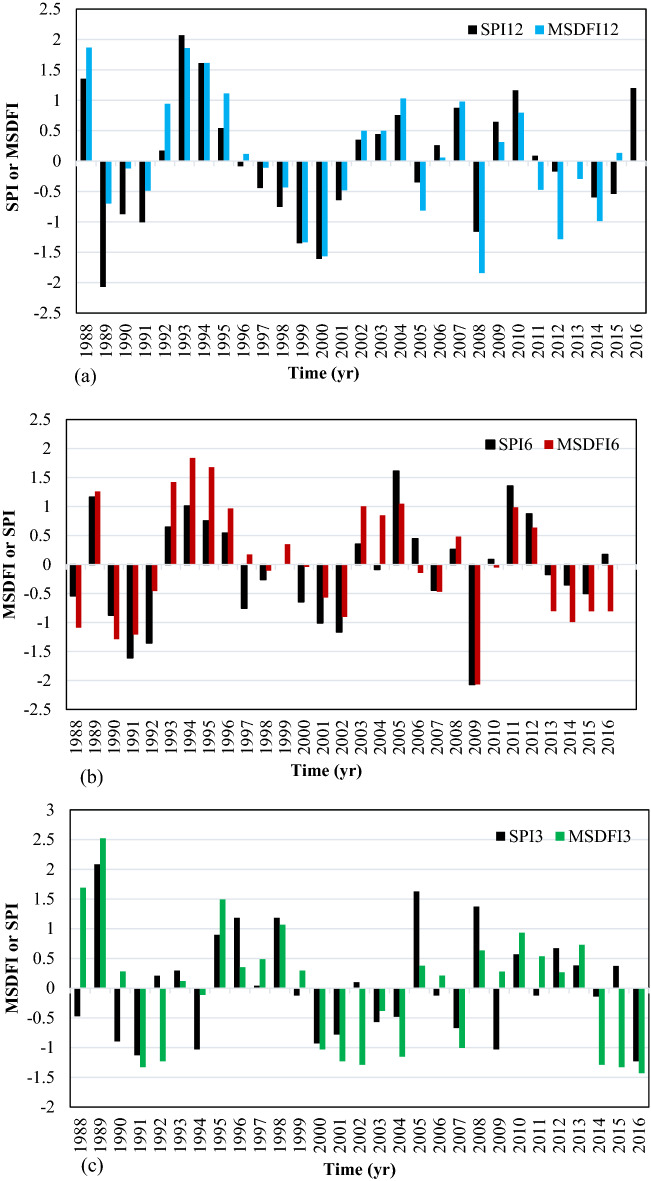
SPI and MSDFI histogram for aggregated (**a**) 12 months ended in September, (**b**) 6 months ended in March, and (**c**) 3 months ended in June in SSB.

Based on SPI12, six severe drought periods and five severe wet conditions have happened during this period. It means that the catchment experienced a severe drought on average every 5 years. A prolonged drought happened from 1996 to 2002. It is important to note that no severe drought has been experienced since 2009. In this period, several moderate droughts happen; however, more frequent wet conditions are observed. It is while much news about the drought and its impacts had been spread in the media during this period in Iran. This issue can be well justified using the alternative MSDFI. The plot of MSDFI12 shows that the catchment experienced three severe wet conditions and four severe drought conditions from 1986 to 2016. It is worth mentioning that one of those severe droughts was felt in 2014 and the drought feeling in 2012 with MSDFI =  − 1.49 was very close to the severe drought class. A comparison of SPI12 and MSDFI12 from 2009 to 2016 shows that the SSB has experienced some moderate drought feeling; although, SPI and precipitation trends do not show any considerable drought. From Fig. 5b,c, it is clear that in the longer period from 1986 to 2004, MSDFI is a little greater than SPI, while the situation is inverse from 2005 to the end of the period. In Fig. 5c the most severe drought is seen in 2014 with SPI3 =  − 2.06 and MSDFI3 =  − 3.12. In this year although the meteorological drought is severe, the sense of drought is much more severe. According to Fig. 5c, from 2004 the frequency of severe drought feeling has increased. The main point is that the absolute difference between MSDFI and SPI at the end of the period is greater than that in the period onset. For better judgment, the histogram of SPI12 and MSDFI12 for September which is the end of the water year in Iran, SPI6 and MSDFI6 for March, and SPI3 and MSDFI3 for June were plotted in Fig. 6.

In Fig. 6a, from 1987 to 2004, SPI shows more severe drought events where MSDFI values are greater than SPI. It means that during this period, sufficient water has been supplied for the population, although hydro meteorologically several drought periods had taken place. For example, an exceptional drought event occurred in 1989 based on the SPI12, while MSDFI12 is about − 0.5 which means that a normal drought was sensed. After 2004, the condition has been completely changed. During this period, more severe water shortages have been felt and whatever time goes on, the difference between SPI12 and MSDFI12 gets larger. In 2008, SPI12 =  − 1.2 while MSDFI12 =  − 1.8 which means a severe drought was sensed while the meteorological condition shows a moderate drought. In reverse, in 2016, SPI12 = 1.25 reports a moderate wet condition while MSDFI12 = 0 indicates a feeling of normal condition. It is a significant difference between the climate condition and the social drought feeling according to the sub-basin water demand. The same condition governs the SPI6-MSDFI6 and SPI3-MSDFI3. Based on Fig. 6b, the MSDFI reaches higher values in wet conditions compared to the SPI and lower values in drought periods up to 2004. As time goes on, the difference between SPI and MSDFI gets lower till 2004 when the time series of SPI and MSDFI matched. After 2004, the MSDFI values got lower in wet conditions and higher in drought periods. It shows that the meteorological drought condition and sense of drought during this period coincided. So, it is possible to say that due to the increasing population and constant precipitation in this sub-basin, a severe water shortage was sensed after 2004.

### Drought analysis in Roshandeh sub-basin (RSB)

**Figure 7:**
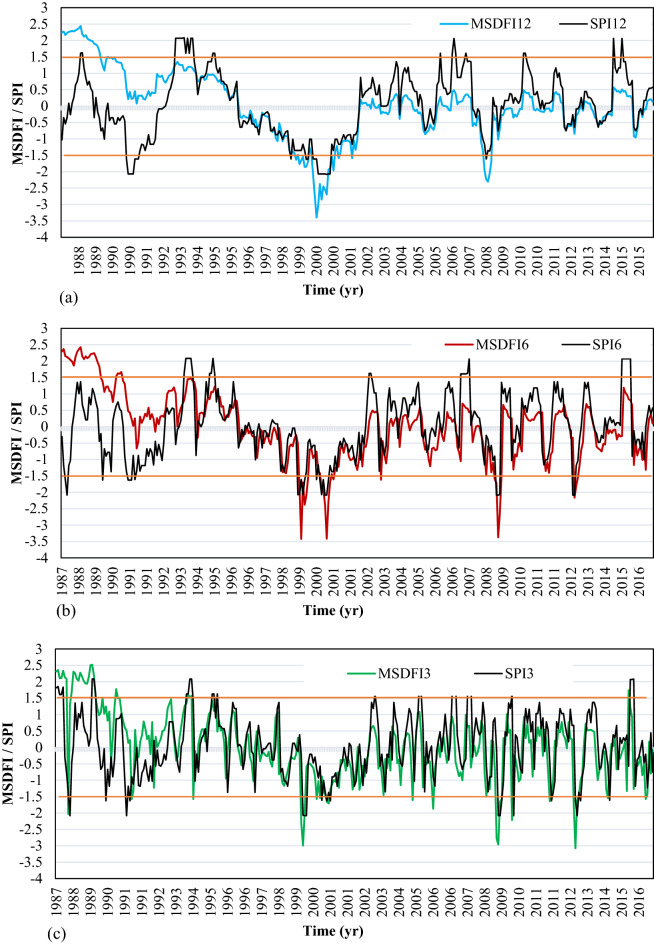
SPI and MSDFI time series in (**a**) 12, (**b**) 6, and (**c**) 3 aggregated months for RSB.

RSB is located at the west of SSB with this difference that it doesn’t contain any populated cities but population growth from 2001 (Fig. 2). For evaluating the drought feeling in this sub-basin, SPI3, SPI6, SPI12, MSDFI3, MSDFI6, and MSDFI12 time series were calculated and plotted in Fig. 7a–c. According to the SPI12 plot, 5 periods of severe drought and 9 periods of severe wet conditions have occurred in this sub-basin. It means that on average, severe droughts occur every 5 years in RSB. The SPI and MSDFI trends in RSB show a different pattern. Due to low population, from 1988 to 1993 there is a considerable difference between the SPI12 and MSDFI12 in both drought and wet periods. The SPI12 values showed a severe wet condition in 1988 while MSDFI indicated a sense of extreme wet condition. Also, during 1990–1991, SPI12 values are less than zero which shows normal to moderate conditions while according to MSDFI12 a moderate wet condition was felt in the sub-basin. The most important distinction between SPI and MSDFI is visible in 1992. As SPI12 =  − 2 which means an extreme drought, MSDFI is above zero which shows a normal feeling condition. It is while, after 1993 till 2002, in all drought periods, MSDFI12 indicates almost similar conditions. However, from 2002, the difference between the SPI12 and MSDFI12 is grown. This is in agreement with the population trend of this sub-basin. During the first 5 years of this period (1988–2016), the population of RSB was very low and even extreme drought did not make a water shortage feeling. However, the population growth rate led to an extreme increase in population respected to 1986. Therefore, water demand increased drastically which led to water stress and drought feeling. Because of high population growth in the last years, the severe wet hydrometeorological conditions from 2002 to 2016 were felt as a normal condition in the sub-basin.

According to Fig. 7, SPI3 and MSDFI3 are in good agreement with those of the 6 and 12 months time scales. However, the same pattern of 6 and 12 months time scales was governed to SPI3 and MSDFI3. Among 9 severe drought periods detected by SPI6, the MSDFI6 detected 4 of them, all with higher severity. Also, among 14 severe drought periods based on the SPI3, 10 of them were detected by MSDFI3, however, four other senses of severe drought are observed in Fig. 7c.

**Figure 8:**
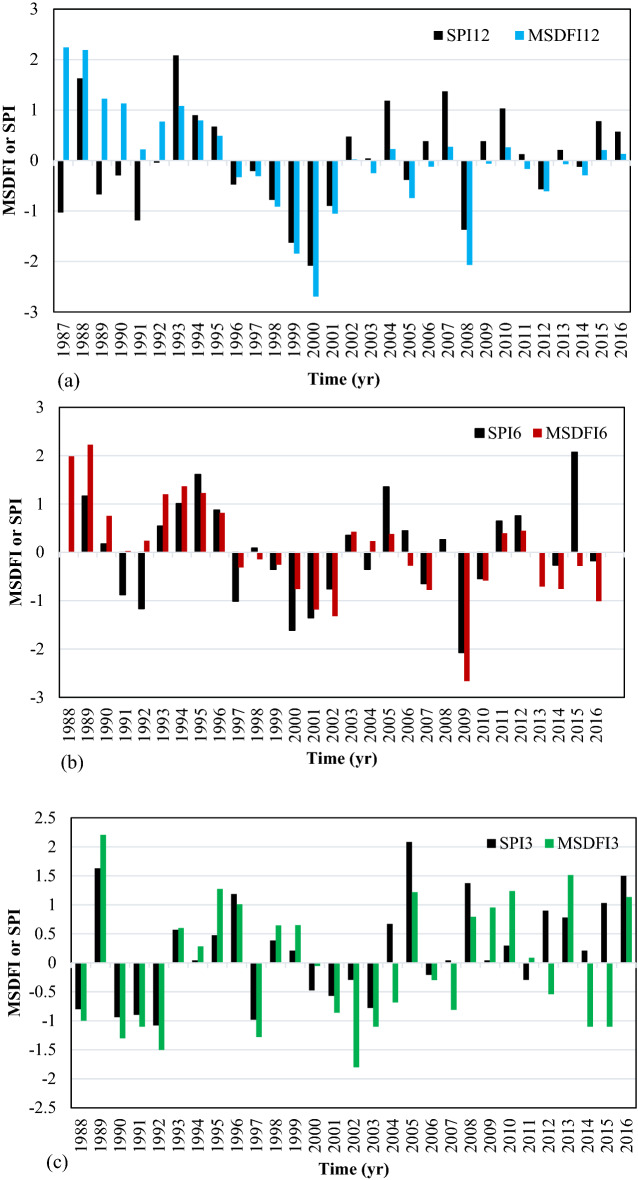
SPI and MSDFI histogram for aggregated (**a**) 12 months ended in September, (**b**) 6 months ended in March, and (**c**) 3 months ended in June for RSB.

The histograms of SPI and MSDFI in different time scales have been plotted in Fig. 8. Based on this figure, for 12-month time scale, the MSDFI tends to be below zero after 1994. Where three moderate, one severe, and one extreme drought period were detected by SPI after 1994, MSDFI showed one moderate, one severe, and two extreme drought conditions from 1995. It is worth noting that in this time interval, three wet conditions could be distinguished according to SPI12, but MSDFI showed no feeling of wet condition. This pattern was governed in the 6-month and 3-month time scales.

### Drought analysis in Up-Zolachai sub-basin (UZSB)

UZSB has located upstream of the Zolachai Basin. It includes 32 villages with a total population of 10,783 people in 2016 (Fig. 2). This population is about 6% of the basin’s total population. In 1986 the population of UZSB was 6880 people which increased to 12,409 by 2006. It means that the annual population growth had been 4.23%. After 2006, the increasing trend changed to a decreasing growth (− 1.31%) such that in 2016 the population reached 10,783 people. Analysis of the precipitation trend (Fig. 3c) shows that precipitation has decreased during the three last decades at a rate of − 1.56%.

**Figure 9:**
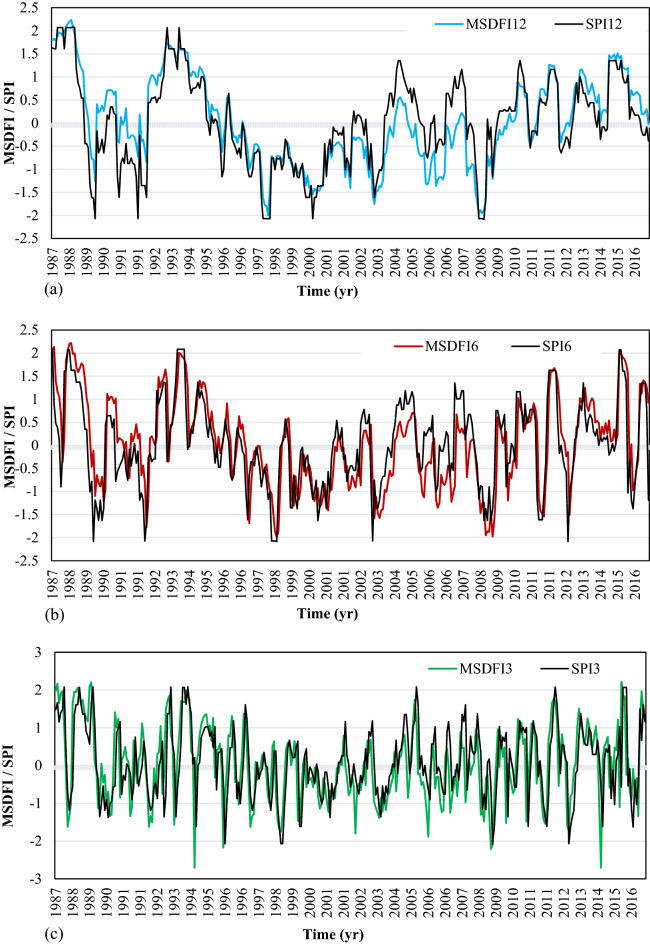
SPI and MSDFI time series in (**a**) 12, (**b**) 6, and (**c**) 3 aggregated months for UZSB.

The time series of SPI and MSDFI in 3, 6, and 12-month time scales in Up-Zolachai catchment were plotted in Fig. 9. According to SPI12 and MSDFI12, during 1987–1997, the MSDFI values were higher than the SPI values. From 1998 to 2001, both SPI and MSDFI matched which shows the water supply and water demand in this period had been balanced. From 2001 up to 2010, the MSDFI values were lower than the SPI which means the water demand overcame the water supply. In this period the population meets the highest value in the sub-basin. Again, from 2010 the MSDFI values outpaced the SPI values. It is due to the decreasing population trend in this period as a result of the significant migration of the inhabitants to the larger cities at downstream sub-basins. The same pattern is visible in 3 and 6 months time scales.

**Figure 10:**
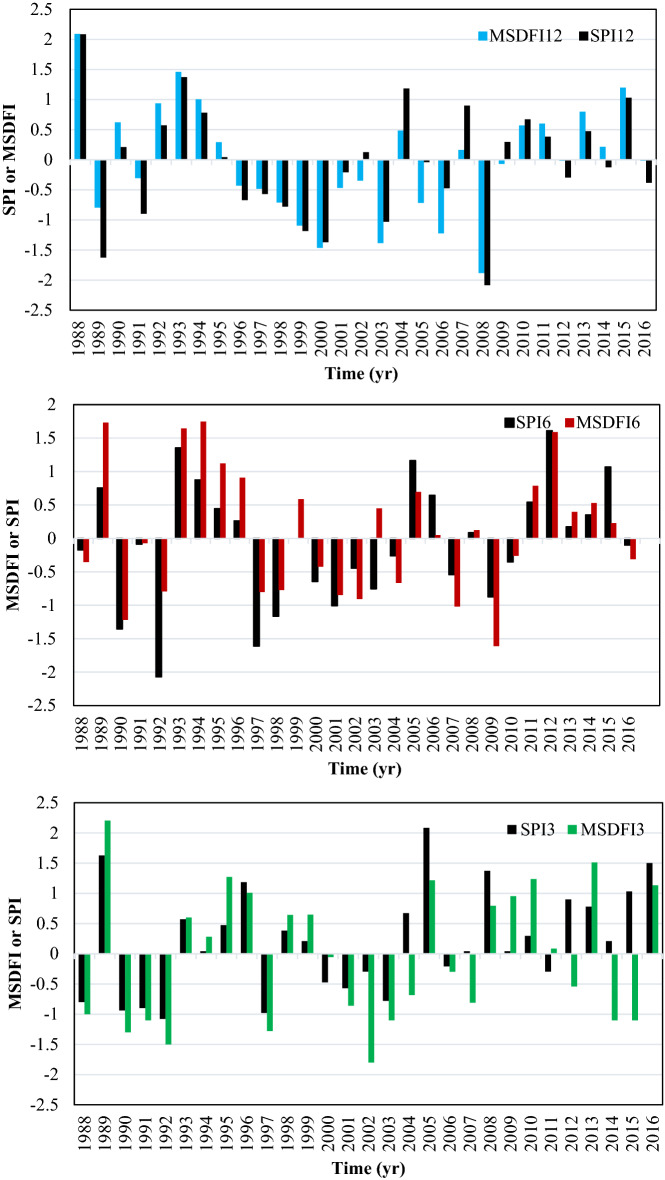
SPI and MSDFI histogram for aggregated (**a**) 12 months ended in September, (**b**) 6 months ended in March, and (**c**) 3 months ended in June for UZSB.

According to Fig. 10, in 12 months time scale, both SPI and MSDFI captured the normal condition in the same way and the SPI and MSDFI values in the normal condition were almost the same. Also, the extreme and severe wet conditions were determined with small differences. However, the severe drought captured by SPI in 1989 was associated with normal conditions determined by MSDFI. It means that although the water supply had reduced significantly in 1989, the drought feeling was not considerable because of low water demand. The same pattern is visible for 6 months time scale in normal conditions. There are two severe drought periods based on SPI6 in 1992 and 1997 but MSDFI6 showed the normal condition. In reverse, in 2009, a normal condition based on SPI6 had a severe drought feeling based on the MSDFI6.

### IDF analysis

**Figure 11:**
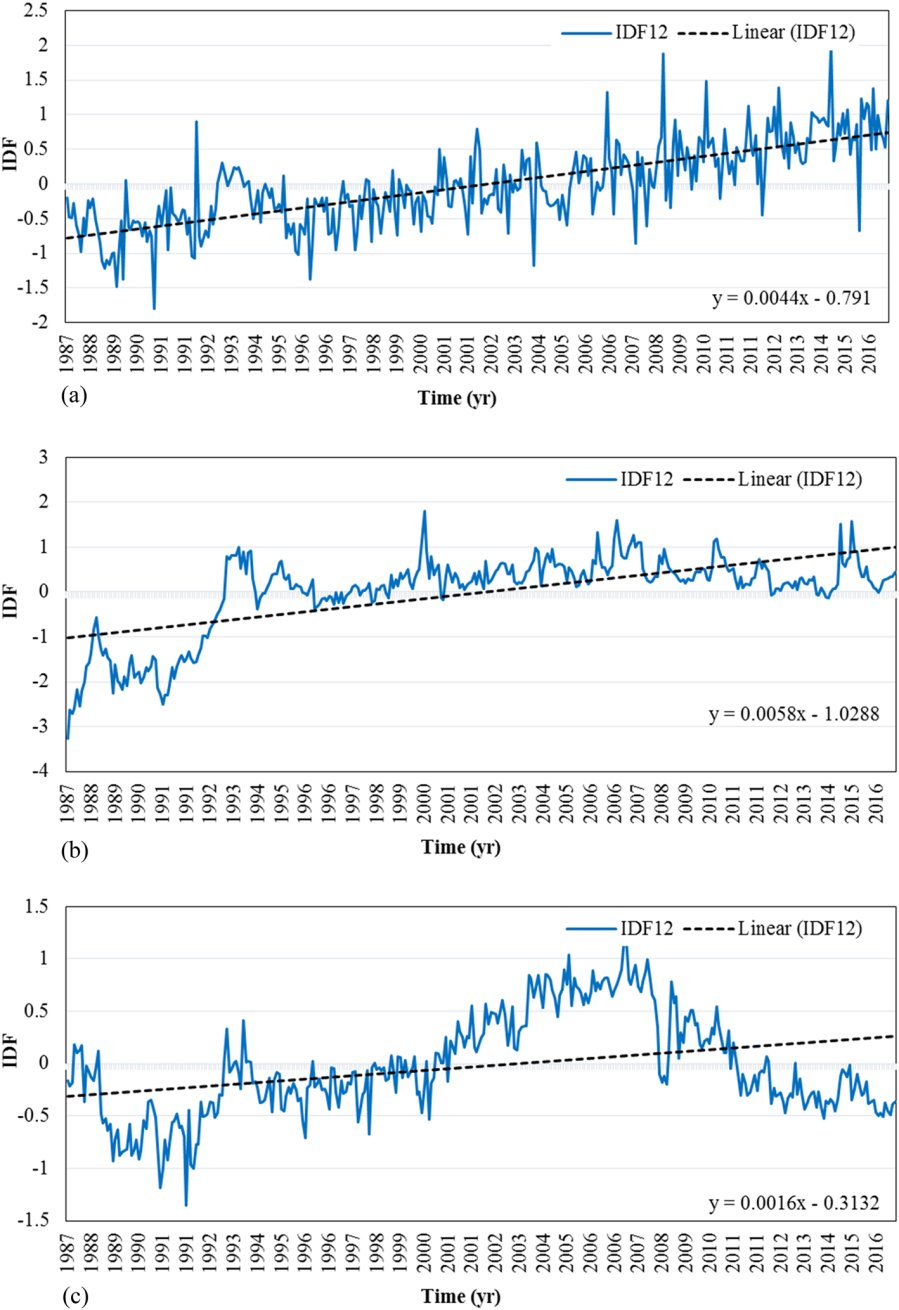
IDF12 time series in (**a**) SSB, (**b**) RSB, and (**c**) UZSB.

To compare the intensity of drought feeling in the upstream and downstream sub-basins, the IDF index in up-Zolachai, Salmas, and Roshandeh sub-basins was calculated using Eq. (3). IDF can be calculated for different time scales, but here just IDF12 is presented (Fig. 11). The trend of IDF6 and IDF3 is similar to IDF12, but IDF12 was selected here because its graph is smoother and shows a clearer trend. As can be seen, the feeling of drought is related to the trend of the population (Fig. 2). According to Fig. 11a with the increase in the population of SSB, the intensity of the feeling of drought has increased. Indeed, the drought and the low economic value of agricultural products in the UZSB and RSB have caused people to migrate downstream for a better life. Therefore, by decreasing the population, the intensity of drought feeling in these sub-basins has declined since 2007 (Fig. 11b,c); although after a severe drought (2008), the UZSB has undergone a mild wet condition. IDF12 time series in this basin (Fig. 11c) follows three distinct parts. The first ends in 2001 in which IDF12 is almost without considerable trend. Then up to 2007, an increasing trend is observed. Finally, the IDF12 falls gradually till 2016. These periods are generally accompanied by the population trend in the sub-basin, while the precipitation variation cannot be neglected, as well.

In contrast, in the SSB, with a large resident population, the feeling of drought has increased and is still increasing. In this sub-basin, although according to the SPI index, severe wet periods have occurred in recent years, the intensity of drought feeling has not decreased significantly. As can be seen in Fig. 11b, the increasing trend of IDF in the RSB stopped in 2004. This trend confirms the hypothesis of this study to differentiate the drought feeling from meteorological drought. In this catchment, although a rapid IDF growth is observed in the first years of the study period, the intensity of drought has been almost constant since 1992. A comparison between SPI12 (Fig. 7a) and IDF12 (Fig. 11b) illustrates that although SPI12 fluctuates between wet and dry months from 1992 to 2016, the sense of drought is almost positive which means in most of the years there has been a sense of drought among the habitants of the sub-basin.

## Discussion

Although many researchers have suggested long-time SPI for socio-economic drought monitoring, it seems that assessing drought by considering only one parameter may not be a good idea. It has been mentioned that the SPI cannot expeditiously relate to precipitation deficit, drought probability, and temporal evolution of the droughts^24,25^. Enhanced severity and duration of drought and its consequences in the recent decades are on one hand due to slight changes in precipitation and air temperature^26^ and on the other hand due to the increased rate of demand for water in the societies^27^. The latter has been the most important factor in the depletion of groundwater resources^28^ and the degradation of aquatic ecosystems like lakes and lagoons^29^. The enhanced water demand has brought a general drought feeling such that every meteorological drought causes extensive concern and deep stress among people and water resources managers. Reports show that the annual per capita amount of renewable water resources in Iran has passed the limit of water stress (based on the Falkenmark water scarcity index), and this is mainly due to the increase in the population rather than the decline in the country’s renewable water resources. Therefore, given the population growth in Iran and consequently the increase in water consumption in recent decades (increased demand), in this study, two effective variables: precipitation as an indicator of water supply and population as an indicator for water demand were employed to better assess the drought. The population can be the indicator of all demand because by increasing the population, the domestic agricultural and industrial water demand will be increased. In some cases, the drought indices show the drought condition while this is not due to the lack of precipitation. It is due to the population growth and increasing demand. So, it is needed to differentiate between drought events and drought feelings. This is the case that motivated the authors to propose a new drought index. Doing so, a meaningful standardized quantitative and qualitative description of the drought was introduced as “multivariate standardized drought feeling index” (MSDFI) and the results were compared with the univariate SPI index which considers only the precipitation variable.

Since the per capita precipitation is taken into account in the MSDFI index not only the amount of climatic precipitation, MSDFI fluctuates with population changes. Therefore, it can be used in drought analysis in areas with severe population changes. Based on the results obtained in this paper, areas that are the destination of immigrants, such as big cities in a basin, may face more social challenges when meteorological drought occurs. Therefore, the issue of drought in these areas is much more sensitive and needs more to be paid attention. But in sparsely populated areas, such as rural areas upstream of basins, even in times of meteorological drought, water scarcity and drought feeling are not so worrisome because the needs of the limited population living in such areas may be easily met. These differences are the main reason that today, in the densely populated areas of the world, the issue of drought is always a major concern among managers and scientists. In these areas, even if severe or continuous droughts do not occur, there is concern about socio-economic damage. Therefore, the issue of drought has always been raised as a challenge in the community and it is necessary to pay attention to the strategies and solutions for facing and adapting to drought.

Based on the IDF index, the severity of the feeling of the drought as the difference between SPI and MSDFI was calculated in different regions of the Zolachai basin. According to the results, in areas where the population is growing rapidly, the IDF has an upward trend. In the study area of this article, the maximum value obtained for this index was about 2, which of course can approach larger values in areas with more population and less rainfall. The trend of IDF shows the changes in water per capita over time and its relationship with the feeling of drought experienced by the residents. This index can also be the basis for analyzing the socio-economic consequences of development in different regions.

One of the limitations of this paper is the lack of population data in a short time scale. In Iran, a population census is done every 5 years. Therefore, short-term population changes are not seen in the calculation of the drought index. Also, residents’ movement among different regions that may be repeated every month or year, although does not significantly influence the results, has been neglected. Moreover, in this study, the population was considered as a simplifying assumption for water consumption because the calculation of actual water consumption and water demands with the available uncertainties is very complicated. Obviously, if real consumption data are available in short-term time scales in a region, it is recommended to use them instead of the population parameter.

## Conclusion

Although the SPI has been widely used for drought assessment worldwide, it has several limitations that make the use of the index more challenging. Drought is a multi-dimensional phenomenon that can not be interpreted just by one parameter. Drought not only affects the weather condition, hydrologic flows, and agricultural productivity, but also the normal life of people in the societies. Therefore, a more comprehensive index is required for assessing the drought consequences. Our review showed that there is a significant difference between the meteorological drought in a region and what is felt by residents. Therefore, to have an effective plan for drought adaptation, separate strategies for drought and the sense of drought should be provided. To quantify the drought feeling, MSDFI was introduced in the current study. The MSDFI is a meaningful index for socio-economic drought assessment when it is used associated with a drought index like SPI. Therefore, it can cover the shortcomings of the meteorological drought indices. As MSDFI captures both supply and water demand it can provide more information about the socio-economic drought. The suggested framework can be appropriately applied in any region where rapid population growth exacerbates the pressure on water resources. MSDFI together with SPI provides complementary information on socio-economic drought development based on the water demand of the population who live in the basin. The IDF index that was defined in this study as the difference between MSDFI and SPI, depicted the increasing trend of drought feeling in the populated areas because of the enhanced water demand while the natural supply does not usually change considerably. On contrary, because of immigration from small residential areas to big cities or their low population growth, the IDF trend is not increasing in such areas, therefore they may have a lower sense of drought consequences.

## Data Availability

The datasets generated during and/or analysed during the current study are available from the corresponding author on reasonable request.
